# Should pediatric parenteral nutrition be individualized?☆

**DOI:** 10.1016/j.rpped.2014.06.006

**Published:** 2014-12

**Authors:** Renata Germano Borges de Oliveira Nascimento Freitas, Roberto José Negrão Nogueira, Margareth Lopes Galvão Saron, Alexandre Esteves Souza Lima, Gabriel Hessel

**Affiliations:** a Universidade Estadual de Campinas (UNICAMP), Campinas, SP, Brazil; b Centro Universitário de Volta Redonda, Volta Redonda, RJ, Brazil

**Keywords:** Nutritional status, Child, Parenteral nutrition

## Abstract

**INTRODUCTION:**

Parenteral nutrition (PN) formulations are commonly individualized, since their standardization appears inadequate for the pediatric population. This study aimed to evaluate the nutritional state and the reasons for PN individualization in pediatric patients using PN, hospitalized in a tertiary hospital in Campinas, São Paulo.

**METHODS:**

This longitudinal study comprised patients using PN followed by up to 67 days. Nutritional status was classified according to the criteria established by the World Health Organization (WHO) (2006) and WHO (2007). The levels of the following elements in blood were analyzed: sodium, potassium, ionized calcium, chloride, magnesium, inorganic phosphorus, and triglycerides (TGL). Among the criteria for individualization, the following were considered undeniable: significant reduction in blood levels of potassium (<3mEq/L), sodium (<125mEq/L), magnesium (<1mEq/L), phosphorus (<1.5mEq/L), ionic calcium (<1mmol), and chloride (<90mEq/L), or any value above the references.

**RESULTS:**

Twelve pediatric patients aged 1 month to 15 years were studied (49 individualizations). Most patients were classified as malnourished. It was observed that 74/254 (29.2%) of examinations demanded individualized PN for indubitable reasons.

**CONCLUSION:**

The nutritional state of patients was considered critical in most cases. Thus, the individualization performed in the beginning of PN for energy protein adequacy was indispensable. In addition, the individualized PN was indispensable in at least 29.2% of PN for correction of alterations found in biochemical parameters.

## Introduction

In 1968, Dudrick, Vars, and Rhoads^1^ became famous for starting parenteral nutrition (PN) formulations.^2^ In 1972, Solassol and Joyeux reported the successful use of the parenteral formula that would become known as 3-in-1, containing amino acids, a glucose and lipid emulsion, as well as electrolytes, vitamins, and trace elements.^3^ PN is an effective nutritional strategy for survival, but it is associated with clinical complications such as infections, metabolic and minerals disorders, hypertriglyceridemia, and liver alterations.^4^ According to the American Society for Parenteral & Enteral Nutrition (ASPEN),^5^ PN is a complex therapy associated with adverse effects, and even death, when safety guidelines are not followed. Thus, for an appropriate and safe prescription, it is necessary to meet the needs of protein, energy, macronutrients, micronutrients, fluid homeostasis, and acid-base balance. 

The PN formula can be standardized or individualized for adults. Regarding the pediatric population, the formulations are commonly individualized due to peculiarities related to growth and development and, consequently, different nutritional demands. However, there have been an increasing number of studies on standardized 3-in-1 PN (industrialized) for children. According to Colomb *et al* 6 and Rigo *et al*,^7^ the advantages of using the standardized solution are: reduction in the risk of infection, decrease in prescription errors and complications caused by inadequate use of incompatible compounds, and easy handling reported by health professionals.

Considering that, in Brazil, the use of standardized PN is not a common practice in Pediatrics, and with regard to the abovementioned facts, the aim of the present study was to evaluate the nutritional status and the reasons for PN individualization in pediatric patients receiving PN in a tertiary hospital in Campinas-SP. 

## Method

This was a longitudinal study performed in 12 patients receiving PN, admitted to the pediatric ward and the pediatric intensive care unit (PICU) of a tertiary hospital in Campinas-SP. Patients were followed for up to 67 days of PN use.

The study inclusion criteria were: use of individualized PN, and signature of the informed consent by the parents/guardians. When the PN was discontinued, but the patient subsequently started receiving it again, this patient was included in the study only with regard to the first instance.

The weight and height of patients were measured according to the techniques proposed by the World Health Organization (WHO)^8^ and Lohman, Roche, and Martorell.9 The instruments used were: stadiometer (to the nearest 0.1cm), electronic Filizola scale (Filizola(r) - São Paulo, Brazil) (capacity of 2.5kg to 150kg), and Toledo digital scale (Toledo(r) - São Paulo, Brazil ) (capacity of 0.1kg to 15kg).

Nutritional status was classified according to the criteria proposed by the WHO^10^^,^ 11 as follows: 

- For the variable weight/age: z-score <-3 is equivalent to very low weight for age; z-score between -3 and -2 is equivalent to low weight for age; z-score is ≥-2 and ≤+2 is equivalent to adequate weight for age; z-score >+2 is equivalent to high weight for age. This variable was used to classify the nutritional status of patients younger than 10 years of age.

- For the variable BMI/age: z-score < -3 is equivalent to severe underweight; z-score between -3 and -2 is equivalent to underweight; z-score ≥-2 and ≤+1 is within the normal range; z-score between +1 and +2 is equivalent to overweight; z-score between +2 and +3 is equivalent to obesity; z-score is > +3 is equivalent to severe obesity. This variable was used to classify the nutritional status of patients older than 10 years of age and was not been applied to patients younger than 10 years due to the difficulty of data collection for height.

To monitor patients receiving PN, it is the routine of the nutrition support team service to perform laboratory tests, as recommended by ASPEN.^2^^-^ 12 Study data included those of up to 24 prior to individualization or re-individualization of the PN bag. Blood measurements of sodium, potassium, ionized calcium, chloride (method: ion selective electrode), magnesium (method: colorimetric xilidil blue), inorganic phosphorus (method: UV phosphomolybdate), and triglycerides (method: colorimetric enzyme) were performed at the Clinical Pathology Laboratory of Hospital de Clínicas, a tertiary hospital in Campinas. Table 1 shows the reference values ​​used by the Clinical Pathology Laboratory.

**Table 1:**
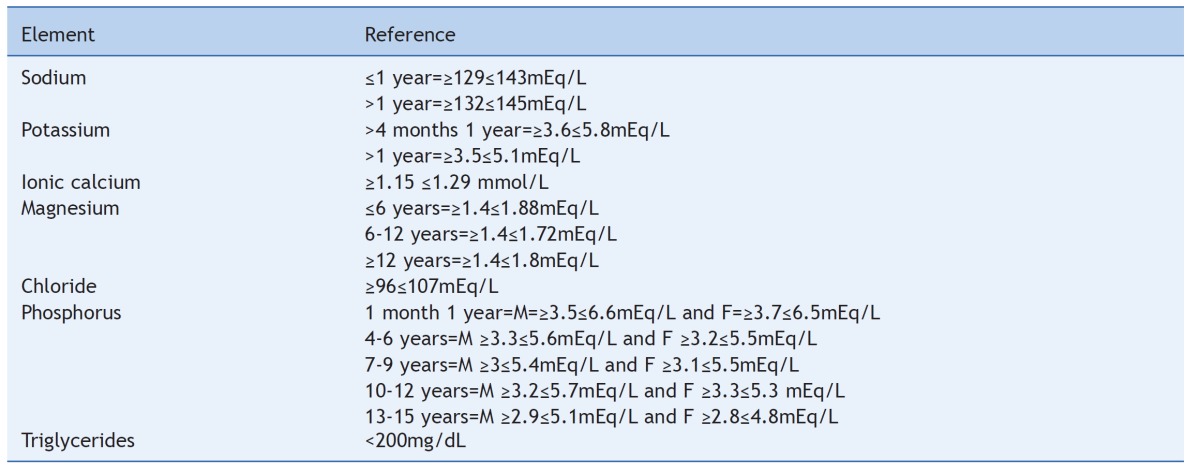
Reference values used by the Laboratory of Clinical Pathology, Hospital de Clinícas, Universidade Estadual de Campinas, Campinas, Brazil.

The criteria used for individualization of PN were: protein-energy adequacy, performed when the patient starts the PN; Protein-energy re-adequacy, performed after the start of the PN; hyponatremia; hypernatremia; hypokalemia; hyperkalemia; hypocalcemia; hypercalcemia; hypomagnesemia; hypermagnesemia; hypophosphatemia; hyperphosphatemia; hypochloremia; hyperchloremia; and hypertriglyceridemia. Among the individualization criteria, the following were considered indisputable: significant reduction in blood levels (potassium <3mEq/L, sodium <125mEq/L, magnesium <1mEq/L, phosphorus <1.5mEq/L, ionic calcium <1 mmol/L, chloride <90 mEq/L), or any value higher than the reference value. For higher values, the need for individualization was ​​considered indisputable, as the infusion discontinuation is the first necessary measure. In the case of triglycerides, values >250mg/dL were considered.

The PN was prescribed by a physician specialized in nutrition and a specialist in parenteral and enteral nutrition working in the nutrition support team. The prescribed amounts were generally in agreement with ASPEN^13^ and the European Society for Pediatric Gastroenterology, Hepatology, and Nutrition (ESPGHAN).^14^ However, in some cases there was initial modification, or during evolution, according to the nutritional and laboratory assessment. The pharmacochemical standards were respected in all cases and attested by the pharmacist of the nutrition support team and by the professional in charge of preparing the formulation, according to Board Resolution No. 63, of 07/06/2000.^15^

The SPSS(r) release 17 was used to assess the frequencies and perform the descriptive analysis of the data. 

The project was approved by the Ethics Committee of the School of Medical Sciences, Universidade Estadual de Campinas (No 538/2011). 

## Results

A total of 12 patients (nine males and three females) were studied, with a total of 49 individualizations. The assessed patients' age ranged from 4 months to 15 years and 4 months, with exclusive PN in 39/49 (79.6%).

Table 2 shows the age, nutritional status, time of assessment, the number of individualizations performed for each patient, place of admission, and patient outcome. The nutritional status of patients, according to the weight/age, was rated as very low weight (n=6), low weight (n=1), or adequate weight for age (n=2). Two adolescents were classified as having severe underweight according to BMI/age (Table 2).

**Table 2:**
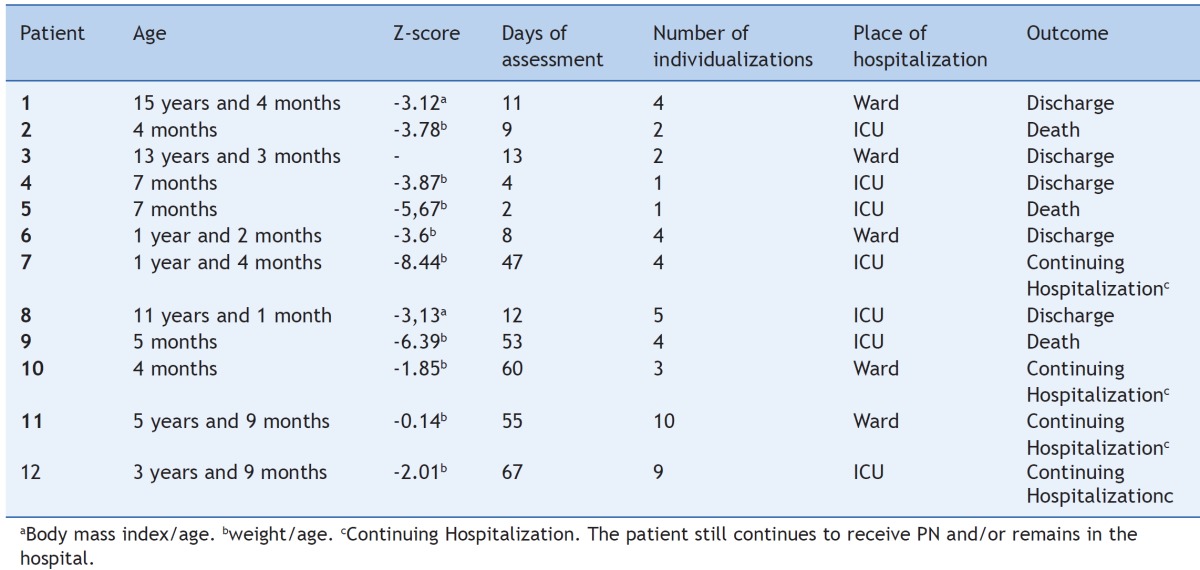
Description of the sample according to age, nutritional status, time of evaluation, PN route of administration, number of individualizations performed in each patient, place of hospitalization, and outcome.

Table 3 shows that, with the exception of ionic calcium and magnesium, laboratory tests were within the reference values ​​in most cases assessed for the 1^st^ individualization. Regarding the re-individualizations, it was observed that magnesium and triglycerides levels were, most of times, out of the reference range. 

**Table 3:**
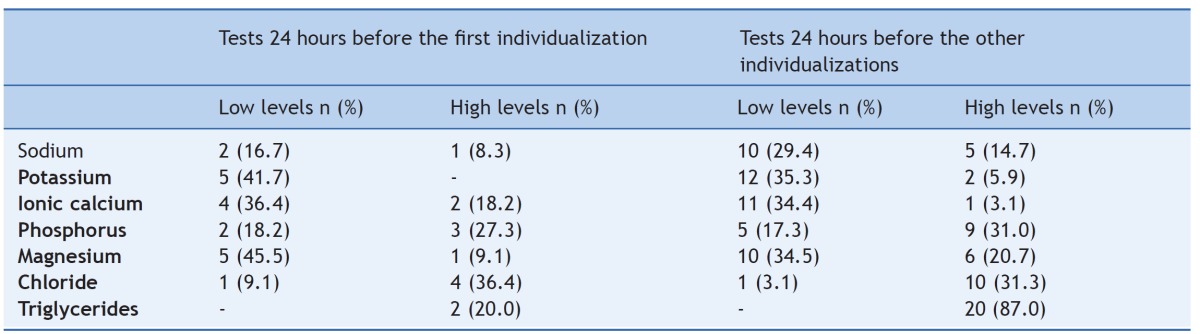
Adequacy of laboratory tests in the 24 hours prior to the first individualization and the other re-individualizations.

Table 4 shows that 74/254 (29.2%) of the assessments required PN individualization due to unquestionable reasons, according to blood levels. 

**Table 4:**
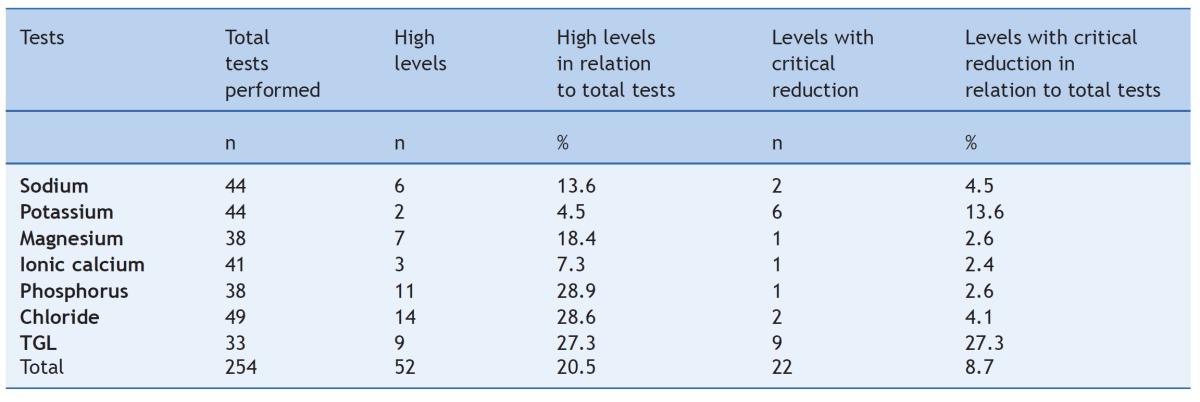
Percentage of unquestionable reasons for the need of individualization, according to blood levels.

At the start of PN use, protein-energy adequacy was observed in accordance with clinical and nutritional status as the only indication or associated to individualization (n=12, 100%). Later, when there was need for re-individualizations, protein-energy readjustment was the main reason in 13/37 (35.2%) cases.

## Discussion

The nutritional status of the hospitalized patient suffers the actions of metabolic stress. Critical pediatric patients, especially infants and children, are very vulnerable to the consequences generated by this situation. During the stress process, there is a loss of fatty tissue as well as muscle, which can aggravate malnutrition and significantly impair response to the disease.

The majority of the assessed patients were admitted to the PICU with exclusive PN, of whom 63.6% were classified as having severe malnutrition. Knowing that body weight may be "masked" due to the inflammatory process with presence of edema, it is possible that the nutritional status of these patients was underestimated, which may raise this malnutrition percentage. Severe malnutrition was found mostly in children aged <1 year and 2 months. This can be explained by the fact that infants are more susceptible to losses of nutritional status in relation to older children. Therefore, the offered nutrition support is of utmost importance for patient survival and recovery.^16^

Nutritional support through PN is one of the ways to improve nutritional status. In pediatrics, PN is commonly individualized; its main advantage is the specific prescription according to the nutritional needs and the clinical condition of patients.^17^ In the present study, at first, the PN of all patients was individualized due to protein-energy adequacy, as the only or associated indication. Later, when re-individualizations were required, protein-energy readjustment was the reason for re-individualizations in 35.2% of cases. However, standardized PN has been identified as a strategic option in some studies.^6^^,^ 7

These studies reported the possible benefits of standardized PN, such as reducing the risk of infection; however, these studies are not comparative. Other advantages mentioned in the studies include the reduction in prescription errors; decrease in complications caused by inadequate use of incompatible compounds; and easy handling, reported by health professionals.^6^^,^ 7

According to the studies by Agostoni^18^ and Colomb *et al*,^6^ most hospitals do not have trained professionals to prescribe PN. Furthermore, few units have adequate conditions to prepare the prescribed PN formula. In Brazil, these conditions are regulated by current legislation (Board Resolution No. 63 of 07/06/2000).^15^

Riskin, Shiff, and Shamir,^17^ verified, through telephone consultations, that of the 25 NICUs in Israel, 18 used standardized PN. Moreover, among the seven NICUs that used individualized PN, six stored standardized PN for weekends and evenings. In Israel, most NICUs are small- and mid-sized and there is no readily available nutrition support team. Thus, the authors suggest the use of standardized PN for most newborns and individualized PN for those who need it. However, according to ASPEN,^19^ in some cases, such as neonates, pediatric, and critically-ill patients, the use of standardized PN can be difficult.

In Brazil, the individualized PN bags are prescribed by the physician and formulated under the supervision of a pharmacist, in accordance with current legislation (Board Resolution No. 63 of 07/06/2000),^15^ which defines the necessary care and controls in the practice of nutritional therapy, including the need of a nutrition support team, which must necessarily be comprised of at least one professional from each category (doctor, nutritionist, nurse, pharmacist, and may include other professional according to the hospital) with specific training.^15^

Moreover, in many places, including Europe and Brazil, computer programs are used to calculate the prescription of individualized PN, helping to prevent the errors mentioned by the authors that argue in favor of standardized PN use, such as technical drug errors. Furthermore, computer programs coordinate the easy handling and convenience of use and communication between the pharmacy and the PN prescription staff.^6^

In the study by Colomb *et al*,^6^ two standard PN formulas (one for term newborns to children aged up to 2 years and another for children aged 2-18 years) were used. Approximately 30% of patients were not included in the study due to the need for individualization. However, the authors state that in clinical practice, these could be included, because the limitations outweigh the risks when the PN is used in the short term. According to ASPEN,^4^ PN formulations are often individualized and standardized PN may be an alternative. According to the ESPGHAN,^14^ a standardized PN formula may be used for short periods (up to two weeks); however, the individualized PN is preferable.^14^^-^ 18

In the present study, one to ten PN individualizations were conducted for each patient during the evaluation period (2-67 days). Although it is not possible to affirm, it can be speculated that PN use for a longer period of time results in a higher cumulative possibility of mineral and TGL disturbances. In fact, the possibility of mineral disturbances is a limiting factor for the prescription of PN. Among the sample patients, 10/12 (83.3%) required at least one re-individualization during PN use. According to all tests performed, 20.5% had values above and 8.7% had values below the reference values, which is considered an unquestionable reason for PN individualization. 

Therefore, 29.2% were considered indisputable. In the study by Krohn *et al*,^19^ PICU patients were evaluated with standardized or individualized PN. The authors noted that 54% of the standardized PN required modifications. That is, it was necessary to carry out PN individualization during its use.

As for laboratory tests performed 24 hours before the first individualization, in this study, most were in agreement with the reference values, ​​except for ionic calcium and magnesium. Subsequently, an increase was observed in the inadequacies (especially TGL, phosphorus, and magnesium). Mineral disorders are common, especially because the sample included severely ill patients. Thus, PN re-individualizations were necessary to control the clinical picture. 

Several studies with patients admitted in PICUs reported changes in levels of magnesium,^21^ potassium,^22^ phosphate,^23^ and ionic calcium,^24^ and the association of these alterations with clinical complications. As for hypertriglyceridemia, it may be associated with the use of PN and metabolic stress, which may be due to the disease or a pre-condition. Regarding the presence of hypomagnesemia, the frequent of use of thiazide diuretics, and especially loop diuretics in critically ill patients, explains its high incidence in this study. In fact, the literature reports a prevalence of 20-70% of hypomagnesemia in critically-ill patients.^21^

Therefore, blood levels of minerals should be monitored frequently and the volume and the adequate amount should be administered in the PN, always assessing the infused medications, the drug compatibility of the solution, and variations resulting from the disease. Thus, the PN for pediatric patients should be individualized and the legislation should be followed, so that the safety and effectiveness of the therapy are guaranteed.

It can be concluded that, in the present study, PN individualizations were essential for an adequate energy-protein intake due to impaired nutritional status and correction of abnormalities found in biochemical tests. Studies with larger samples may or may not confirm these findings in their entirety. Nevertheless, for pediatric patients, there is a clear need for PN individualization prescribed by professionals (the nutrition support team), aided and supervised by the hospital pharmacies. This seems to be the safest and most effective way to prescribe PN. 
