# Supplementary material for: Should pediatric parenteral nutrition be individualized?
Source: Rev Paul Pediatr. 2014 Dec;32(4):326–32. doi: 10.1016/j.rpped.2014.06.006 (PMC4311786; doi:10.1016/j.rpped.2014.06.006)
Supplement: Translation

# Translation: Deve-se individualizar a nutrição parenteral pediátrica?☆

**Authors:** Renata Germano Borges de Oliveira Nascimento Freitas, Roberto José Negrão Nogueira, Margareth Lopes Galvão Saron, Alexandre Esteves Souza Lima, Gabriel Hessel

**Affiliations:** a Universidade Estadual de Campinas (UNICAMP), Campinas, SP, Brasil; b Centro Universitário de Volta Redonda, Volta Redonda, RJ, Brasil

**Keywords:** Estado nutricional, Criança, Nutrição parenteral

## Abstract

**INTRODUÇÃO:**

As formulações da nutrição parenteral (NP) são comumente individualizadas, visto que a padronização destas parece inadequada para a população pediátrica. O objetivo do estudo foi avaliar o estado nutricional e os motivos para individualização da NP dos pacientes pediátricos em uso de NP internados em um hospital terciário de Campinas-SP.

**MÉTODOS:**

Estudo longitudinal conduzido com pacientes acompanhados por até 67 dias de uso de NP. Para a classificação do estado nutricional, foram utilizados os critérios propostos pela *World Health Organization* (WHO) (2006) e WHO (2007). As dosagens sanguíneas analisadas foram: sódio, potássio, cálcio iônico, cloreto, magnésio, fósforo inorgânico e triglicerídeo (TGL). Foram considerados motivos indubitáveis para individualização da NP quando esses elementos apresentavam redução expressiva dos níveis sanguíneos (potássio <3 mEq/L; sódio <125 mEq/L; magnésio <1 mEq/L; fósforo <1,5 mEq/L; cálcio iônico <1 mmol/L; cloreto <90 mEq/L) ou qualquer valor superior aos de referência.

**RESULTADOS:**

Foram estudados 12 pacientes (49 individualizações) com idade de 1 mês a 15 anos. A maioria dos pacientes foi classificada como desnutrida. Observou-se que 74/254 (29,2%) dos exames demandaram NP individualizada por motivos indubitáveis.

**CONCLUSÃO:**

O estado nutricional dos pacientes foi considerado crítico, na maioria dos casos. Desta forma, a individualização realizada no início da NP para a adequação energética proteica foi essencial. Além disto, a NP individualizada foi indispensável em, no mínimo, 29,2% das NP, para correção das alterações encontradas nos exames bioquímicos.

## Introdução

Em 1968, Dudrick, Vars e Rhoads^1^ notabilizaram-se por iniciar as formulações de nutrição parenteral (NP).^2^ Em 1972 foi relatada, pelos pesquisadores Solassol e Joyeux, a utilização com sucesso da fórmula parenteral que ficaria conhecida como 3 em 1, contendo aminoácidos, emulsão lipídica e glicose, além de eletrólitos, vitaminas e oligoelementos.^3^ A NP é uma estratégia nutricional eficaz para sobrevida porém, está associada com complicações clínicas como infecções, distúrbios metabólicos, distúrbios de minerais, hipertrigliceridemia e alterações hepáticas.^4^ De acordo com a American Society for Parenteral & Enteral Nutrition (ASPEN),^5^ a NP é uma terapia complexa associada a efeitos adversos e até à morte, quando as diretrizes de segurança não são seguidas. Desta forma, para uma prescrição adequada e segura, é necessário conhecer as necessidades de proteína, energia, macronutrientes, micronutrientes, homeostase de fluidos e equilíbrio ácido base. 

A fórmula da NP pode ser padronizada ou individualizada para adultos. Com relação à população pediátrica, as formulações são, comumente, individualizadas devido a peculiaridades relacionadas ao crescimento e desenvolvimento e, consequentemente, demandas nutricionais diferentes. Contudo, crescem os estudos sobre NP padronizada (industrializada), 3 em 1, para crianças. Segundo Colomb *et al* 6 e Rigo *et al*,^7^ as vantagens para utilização da solução padronizada são: redução dos riscos de infecção, diminuição dos erros de prescrição e das complicações ocasionadas pelo uso indevido de compostos incompatíveis e facilidade relacionada ao manuseio, relatada por profissionais da saúde.

Considerando que, no Brasi,l a utilização de NP padronizada não é uma prática comum na pediatria e diante do exposto, o objetivo do estudo foi avaliar o estado nutricional e os motivos para individualização da NP dos pacientes pediátricos em uso de NP internados em um hospital terciário de Campinas-SP.

## Método

Este estudo teve caráter longitudinal conduzido com 12 pacientes em uso de NP internados na enfermaria e UTI pediátrica de um hospital terciário de Campinas-SP. Os pacientes foram acompanhados por até 67 dias de uso de NP.

Os critérios para inclusão no estudo foram: utilização da NP individualizada e assinatura do Termo de Consentimento Livre e Esclarecido pelo responsável. Quando o paciente recebia alta da NP, mas retornava ao uso da mesma posteriormente, este paciente foi incluído no estudo apenas pela primeira vez. 

Foram aferidos o peso e a altura dos pacientes segundo as técnicas propostas pela *World Health Organization (WHO)* 8 e Lohman, Roche e Martorell.^9^ Os instrumentos utilizados foram: estadiômetro (com precisão de 0,1cm) e balança digital da marca Filizola(r) eletrô nica (capacidade de 2,5kg a 150kg) e Toledo^(r)^ eletrô nica (capacidade de 0,1kg a 15kg).

O estado nutricional foi classificado, de acordo com os critérios propostos pela WHO^10 ^e WHO^11 ^conforme segue:

- Para a variável peso/idade: se o escore z é menor que -3, equivale a muito baixo peso para a idade; se o escore z se situa entre -3 e -2, equivale a baixo peso para a idade; se o escore z é maior ou igual a -2 e menor ou igual a +2, equivale a peso adequado para a idade; se o escore é maior que +2, equivale a peso elevado para a idade. Esta variável foi utilizada para classificação do estado nutricional de pacientes até 10 anos de idade. 

- Para a variável IMC/idade: se o escore z é menor que -3, isso equivale a magreza acentuada; se o escore z se situa entre -3 e -2, isso equivale a magreza; se o escore z é maior ou igual a -2 e menor ou igual a +1, eutrofia; se o escore se situa entre +1 e +2, isso equivale a sobrepeso; se o escore se encontra entre +2 e +3, isso equivale a obesidade; se o escore é maior que +3, isso equivale a obesidade grave. Esta variável foi utilizada para classificação do estado nutricional de pacientes acima de 10 anos de idade e não foi aplicada para pacientes com menos de 10 anos devido à dificuldade de coleta de dados da altura.

Para acompanhamento do paciente em uso de NP, é rotina do serviço da Equipe Multidisciplinar de Terapia Nutricional (EMTN) a realização de exames laboratoriais, conforme o recomendado pela *ASPEN.* 2 ^-^ 12 Computou-se para o estudo os dados de até 24 horas anteriores à individualização ou à reindividualização da bolsa de NP. Realizaram-se as dosagens sanguíneas de sódio, potássio, cálcio iônico, cloreto (método: Eletrodo Íon Seletivo), magnésio (método: Colorimétrico Azul de Xilidil), fósforo inorgânico (método: fosfomolibidato U.V) e triglicerídeo (TGL) (método: Enzimático colorimétrico) no Laboratório de Patologia Clínica do Hospital de Clínicas de um hospital terciário de Campinas. Na tabela 1 seguem os valores de referência utilizados pelo Laboratório de Patologia Clínica do Hospital de Clínicas.

**Tabela 1:** Valores de referência utilizados pelo Laboratório de Patologia Clínica do Hospital de Clínicas, Campinas, Brasil.

Os critérios utilizados para individualização da NP foram: adequação energética proteica, realizada quando o paciente inicia a NP; readequação energética proteica, realizada após o início da NP; hiponatremia; hipernatremia; hipopotassemia; hiperpotassemia; hipocalcemia; hipercalcemia; hipomagnesemia; hipermagnesemia; hipofosfatemia; hiperfosfatemia; hipocloremia; hipercloremia; hipertrigliceridemia. Dentre os critérios para individualização considerados indubitáveis, destacam-se: redução expressiva dos níveis sanguíneos (potássio <3 mEq/L; sódio <125 mEq/L; magnésio <1 mEq/L; fósforo <1,5 mEq/L; cálcio iônico <1 mmol/L; cloreto <90 mEq/L) ou qualquer valor superior aos de referência. Para os valores superiores considerou-se a necessidade de individualização indubitável, pois a interrupção da infusão é a primeira medida necessária. No caso do TGL foram considerados valores >250 mg/dL.

A NP foi prescrita por um dos médicos nutrólogos e especialista em nutrição parenteral e enteral que trabalham na EMTN. As quantidades prescritas estavam, de modo geral, em concordância com a ASPEN^13 ^e a European Society for Paediatric Gastroenterology Hepatology and Nutrition (ESPGHAN).^14^ Porém, em alguns casos houve modificação inicial ou na evolução, segundo a avalição nutricional e laboratorial. As normas fármacoquímicas foram respeitadas em todos os casos e atestada pelo farmacêutico da EMTN e pela manipuladora destinada para confecção da mesma, de acordo com a Resolução da Diretoria Colegiada nº. 63 de 06/07/2000.^15^

Utilizou-se o programa de computador *Statiscal Package for the Social Sciences*(r) (SPSS) versão 17 para apresentar as frequências e analises descritivas dos dados.

O projeto foi aprovado pelo Comitê de Ética e Pesquisa da Faculdade Ciências Médicas da UNICAMP (N^o ^538/2011).

## Resultados

Foram estudados 12 pacientes (9 meninos e 3 meninas) com um total de 49 individualizações. Os pacientes avaliados apresentaram idade variando de 4 meses a 15 anos e 4 meses de vida, sendo a NP exclusiva em 39/49 (79,6%). 

Na tabela 2, observa-se a idade, o estado nutricional, o tempo de avaliação, a quantidade de individualizações realizadas em cada paciente, o local de internação e o desfecho do paciente. O estado nutricional dos pacientes, de acordo com o peso/idade, foi classificado como: muito baixo peso (n=6), baixo peso (n=1) e peso adequado para idade (n=2). Dois adolescentes foram classificados com magreza acentuada segundo o IMC/idade (tabela 2). 

**Tabela 2:** Descrição da amostra, segundo a idade, o estado nutricional, o tempo de avaliação, a via de acesso da NP, a quantidade de individualizações realizadas em cada paciente, o local de internação e o desfecho

Na tabela 3, observa-se que, com exceção do cálcio iônico e do magnésio, os exames laboratoriais estavam dentro dos valores de referência na maioria dos casos avaliados para a 1ª individualização. Com relação às reindividualizações, percebeu-se que o magnésio e os triglicerídeos estavam, na maioria das vezes, fora dos parâmetros de referência. 

**Tabela 3:** Adequação dos exames laboratoriais nas 24 horas anteriores à 1ª individualização e às demais reindividualizações

Na tabela 4, observa-se que 74/254 (29,2%) dos exames demandaram NP individualizada por motivos indubitáveis, de acordo com os níveis sanguíneos.

**Tabela 4:** Percentual de motivos indubitáveis para necessidade de individualização, de acordo com os níveis sanguíneos

No início da utilização da NP, observou-se a adequação energética proteica de acordo com o estado clínico e nutricional como indicação isolada ou associada para individualização (n=12; 100%). Posteriormente, quando houve necessidade de reindividualizações, a readequação energética proteica foi o motivo principal em 13/37 (35,2%) casos.

## Discussão

O estado nutricional do indivíduo enfermo e hospitalizado sofre as ações do estresse metabólico. Pacientes pediátricos críticos, especialmente lactentes e crianças, são muito vulneráveis às consequências geradas por esta situação. Durante o processo de estresse há uma perda do tecido adiposo e também do muscular, que pode agravar a desnutrição e prejudicar sobremaneira a resposta à doença.

A maioria dos pacientes avaliados estava internada na UTI pediátrica com NP exclusiva, sendo 63,6% classificada como portadora de desnutrição grave. Sabendo que o peso corporal pode estar "mascarado" devido ao processo inflamatório com presença de edema, é possível que o estado nutricional destes pacientes estivesse subestimado, podendo elevar esta porcentagem de desnutrição. A desnutrição grave foi encontrada, principalmente, entre as crianças com idade inferior a 1 ano e 2 meses. Isto pode ser explicado pelo fato dos lactentes serem mais susceptíveis a prejuízos do estado nutricional em relação às crianças mais velhas. Desta forma, o suporte nutricional ofertado é de extrema importância para sobrevida e recuperação do paciente.^16^

O suporte nutricional por via NP é uma das maneiras de melhorar o estado nutricional. Em pediatria esta é comumente individualizada e sua principal vantagem é a prescrição específica de acordo com as necessidades nutricionais e a condição clínica dos pacientes.^17^ No presente estudo, em um primeiro momento, a NP de todos os pacientes foi individualizada devido à adequação energética proteica, como indicação isolada ou associada. Posteriormente, quando houve necessidade de reindividualizações, a readequação energética proteica foi o motivo em 35,2% dos casos. Todavia, a NP padronizada é apontada como uma possibilidade estratégica em alguns trabalhos.^6^^,^ 7 Estes trabalhos referem os possíveis benefícios da NP padronizada, tais como a redução dos riscos de infecção, contudo, estes trabalhos não são comparativos. Outras vantagens mencionadas seriam a redução dos erros de prescrição, a diminuição de complicações ocasionadas pelo uso indevido de compostos incompatíveis e a facilidade relacionada ao manuseio, relatada por profissionais da saúde.^6^^,^ 7

De acordo com os trabalhos de Agostoni^19^ e Colomb *et al*,^6^ a maioria dos hospitais não possui profissionais capacitados para prescrever a NP. Além disto, são poucas as unidades em condições adequadas para preparar a fórmula de NP prescrita. No Brasil estas condições são regulamentadas na legislação vigente (Resolução da Diretoria Colegiada nº. 63 de 06/07/2000).^15^

Riskin, Shiff e Shamir,^17^ constataram, por meio de consulta telefônica que, dentre as 25 UTIs neonatais de Israel, 18 utilizavam NP padronizada. Além disto, entre as sete UTIs neonatais que utilizavam NP individualizada, seis estocavam NP padronizada para finais de semana e período noturno. Em Israel, a maioria das UTIs neonatais é pequena e de médio porte e não há EMTN prontamente disponível. Desta maneira, os autores sugerem a utilização de NP padronizada para a maioria dos neonatos e a NP individualizada para aqueles que necessitarem. Porém, de acordo com a ASPEN,^19^ em alguns casos, como de neonatos, pacientes pediátricos e críticos, a utilização da NP padronizada pode ser difícil.

No Brasil, as bolsas de NP individualizadas são prescritas pelo médico e formuladas sob a supervisão de um farmacêutico de acordo com a legislação vigente (Resolução da Diretoria Colegiada nº. 63 de 06/07/2000),^15^ que define os cuidados e controles necessários na prática de terapia nutricional, inclusive a necessidade da EMTN, que deve ser obrigatoriamente constituída por, no mínimo, um profissional de cada categoria (médico, nutricionista, enfermeiro, farmacêutico e pode incluir outro profissional de acordo com a unidade hospitalar) com treinamento específico.^15^

Ademais, em muitos locais, inclusive da Europa e do Brasil, programas de computador são utilizados para cálculo da prescrição da NP individualizada, contribuindo para que não ocorram os erros mencionados pelos autores que defendem a utilização da NP padronizada, como os erros fármaco técnicos. Além disto, os programas de computador cooperam com a facilidade e praticidade na utilização e comunicação entre a farmácia e a equipe de prescrição da NP^6^.

No trabalho de Colomb *et al*,^6^ foram utilizadas 2 fórmulas de NP padronizadas (uma para recém-nascidos a termo até crianças de 2 anos e outra para crianças de 2 a 18 anos de idade). Cerca de 30% dos pacientes não foram incluídos na pesquisa devido à necessidade de individualização. Contudo, os autores afirmam que na prática clínica estes poderiam ser incluídos porque as limitações encontradas superam os riscos, quando a NP é utilizada em curto prazo. Segundo a ASPEN,^4^ as formulações da NP são frequentemente individualizadas e a NP padronizada pode ser uma alternativa. De acordo com a ESPGHAN,^14^ a fórmula de NP padrão pode ser utilizada por períodos curtos (até 2 semanas), contudo a NP individualizada é preferencial.^14^^-^ 19

No presente estudo, foram realizadas de uma a dez individualizações em cada paciente durante o período de avaliação (2-67 dias. Embora, não seja possível afirmar, pode-se especular que a NP por tempo mais prolongado implica em maior possibilidade cumulativa de distúrbios dos minerais e dos TGL. De fato, a possibilidade de distúrbios de minerais é limitante na prescrição de NP. Entre os pacientes da amostra, 10/12 (83,3%) necessitaram de, no mínimo, uma reindividualização ao longo da utilização da NP. De acordo com todos os exames realizados, 20,5% apresentam valores acima dos valores de referência e 8,7% valores abaixo, sendo considerados motivos indubitáveis para individualização da NP. Assim, 29,2% foram consideradas inquestionáveis. No estudo de Krohn *et al*,^20^ foram avaliados pacientes da UTI pediátrica com NP padronizada ou individualizada. Os autores observaram que 54% das NP padronizadas necessitaram de modificações. Ou seja, foi necessário realizar individualizações ao longo da utilização da NP.

Quanto aos exames laboratoriais realizados 24 horas antes da 1ª individualização, no presente estudo, a maioria estava de acordo com os valores de referência, exceto o cálcio iônico e o magnésio. Em seguida, foi observado um aumento de inadequações (especialmente o TGL, fósforo e magnésio). É comum haver os distúrbios de minerais, especialmente, porque a amostra foi composta de pacientes graves. Logo, houve a necessidade de reindividualizações da NP para controle do quadro clínico. Diversos trabalhos com pacientes pediátricos internados em UTI relatam alterações dos níveis de magnésio,^21^ potássio,^22^ fosfato^23^ e cálcio iônico^24^ e associação destas alterações com complicações clínicas. Já a hipertrigliceridemia pode estar associada com a utilização da NP e ao estresse metabólico, podendo se dever à doença ou condição prévia. Em relação a hipomagnesemia, a presença frequente de utilização de diuréticos tiazídicos e, principalmente, os de alça em pacientes graves explica a incidência grande neste trabalho. De fato, a literatura reporta uma prevalência de 20-70% de hipomagnesemia em pacientes graves.^21^ Desta forma, os níveis sanguíneos dos minerais devem ser monitorados com frequência e o volume e a quantidade adequada devem ser administrados na NP, avaliando, sempre, as medicações infundidas, a fármaco-compatibilidade da solução e as variações decorrentes da doença. Assim, o ideal é que a NP para pacientes pediátricos seja individualizada e que haja cumprimento da Legislação para que a segurança e a eficácia sejam garantidas.

Pode-se concluir que, no presente estudo, as individualizações da NP foram essenciais para a adequação energética proteica devido ao estado nutricional debilitado e para a correção das alterações encontradas nos exames bioquímicos. Estudos com amostras maiores podem corroborar ou não com estes achados na sua plenitude. De qualquer modo, para paciente pediátricos fica clara a necessidade de que a NP seja individualizada por profissionais capacitados (da EMTN), auxiliados e fiscalizados pelas farmácias de manipulação para tal. Esta parece ser a maneira mais segura e eficaz de prescrever a NP.
